# Supplementary material for: Soil Microbial Community Responses to Different Management Strategies in Almond Crop
Source: J Fungi (Basel). 2023 Jan 10;9(1):95. doi: 10.3390/jof9010095 (PMC9864756; doi:10.3390/jof9010095)

Figure S2: Venn diagram with shared and exclude genera. Bacterial (A) and fungal (B) genera present in the three soil replicas across managements.

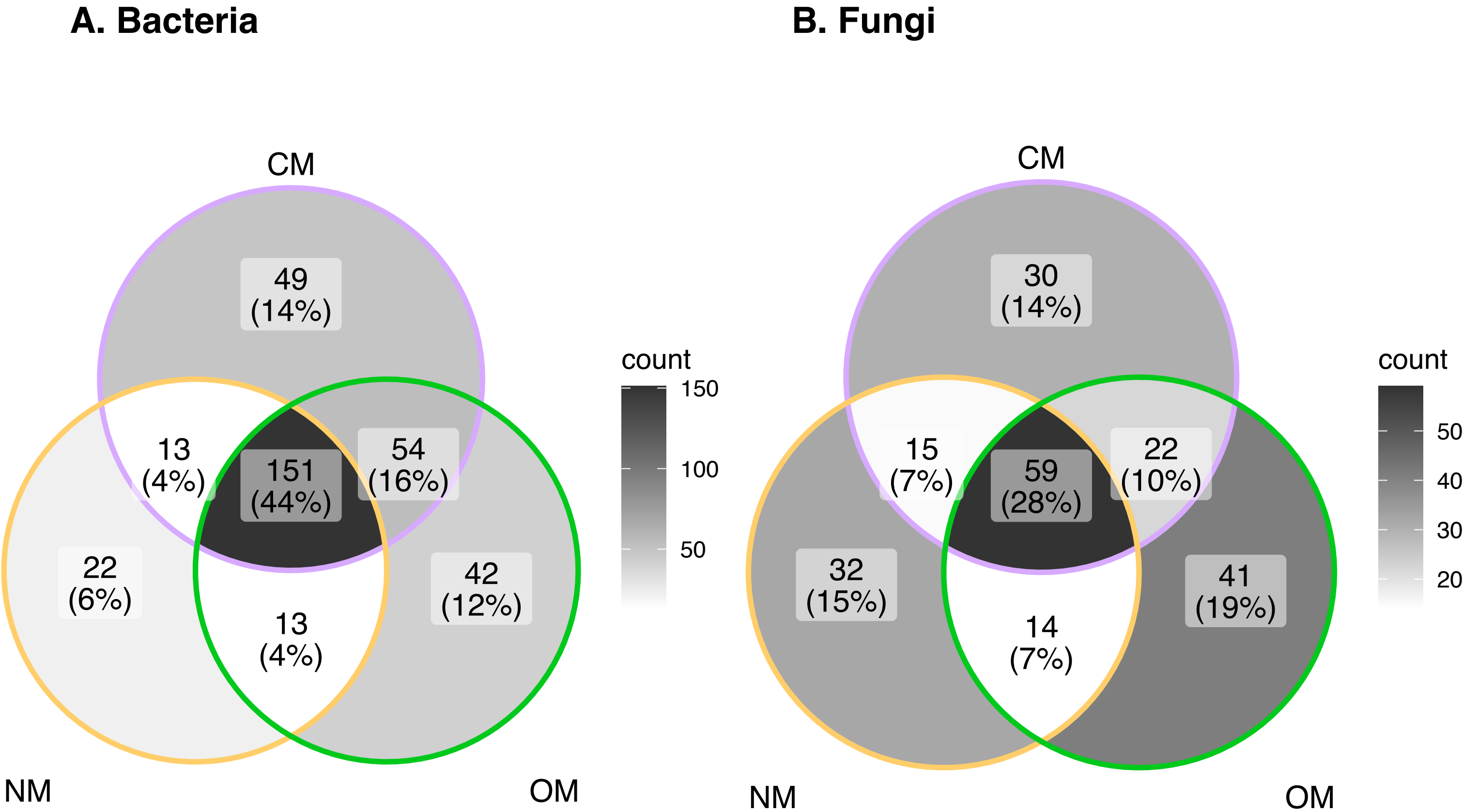

Supplement: Supplementary file 1 [file jof-09-00095-s001.zip › FigureS2_vennDiagram.pdf]
