# Supplementary figures and images for: Soil Microbial Community Responses to Different Management Strategies in Almond Crop
Source: J Fungi (Basel). 2023 Jan 10;9(1):95. doi: 10.3390/jof9010095 (PMC9864756; doi:10.3390/jof9010095)

Figure S1: Rarefaction curves. Rarefaction curves on (A) 16S and (B) ITS datasets.

A

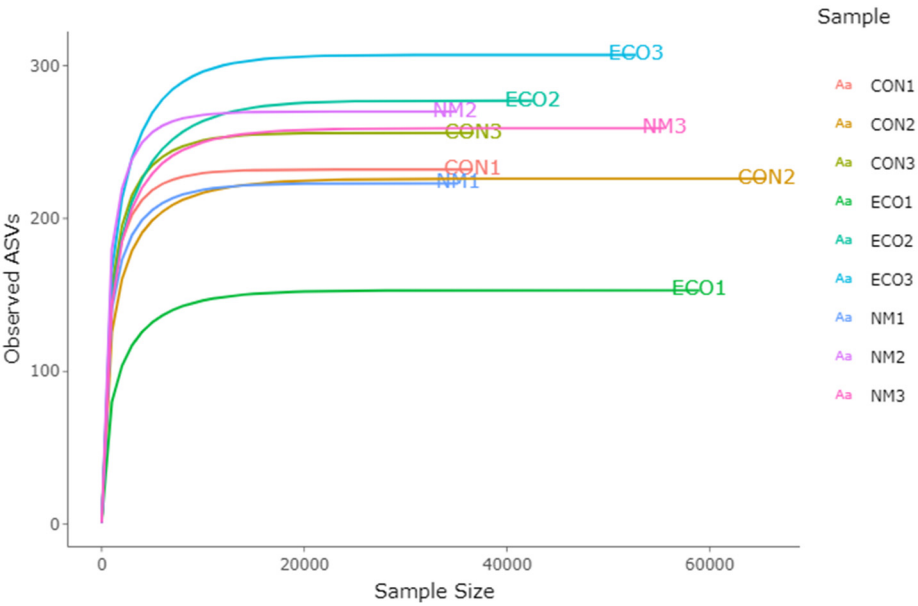

B

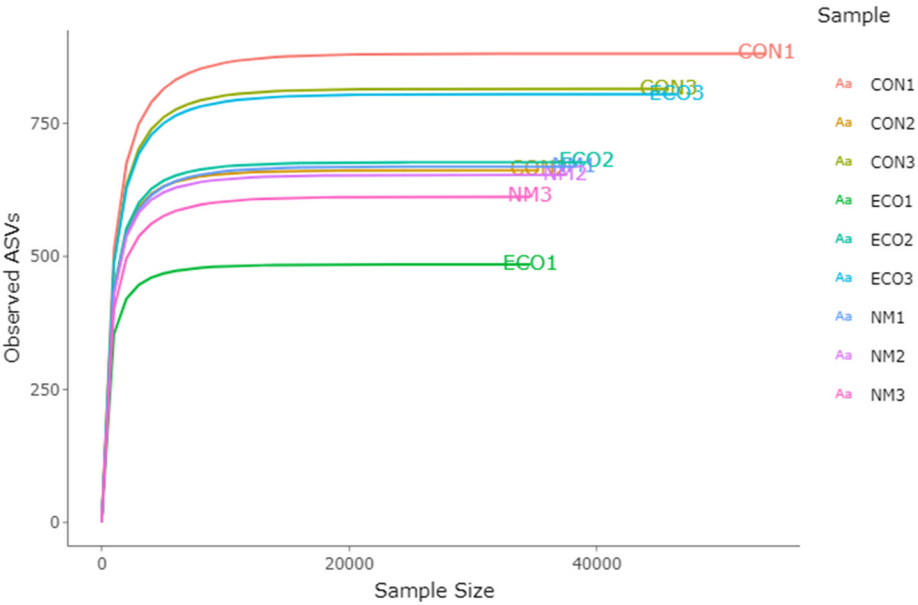

Supplement: Supplementary file 1 [file jof-09-00095-s001.zip › FigureS1.pdf]
